# Supplementary material for: Reproducibility of the German and Slovakian Versions of the Dietary Habits and Nutrition Beliefs Questionnaire (KomPAN)
Source: Nutrients. 2022 Nov 19;14(22):4893. doi: 10.3390/nu14224893 (PMC9696161; doi:10.3390/nu14224893)
Supplement: Supplementary file 1 [file nutrients-14-04893-s001.zip › nutrients-2012028-supplementary.pdf]

Table S1. Nutrition questionnaire according to the number of categories of responses in students from Germany (%)

Question	No. of Cat.	Test							Retest							*p*
		1	2	3	4	5	6	7	1	2	3	4	5	6	7	
DH-Number of meals a day	5	1.3	2.4	55.1	20.0	2.7			1.4	19.3	64.6	11.7	3.1			ns
DH-Regularity of consuming meals	3	32.1	54.9	13.0					34.3	53.6	11.6	0.5				ns
DH-Frequency of snacking between meals	6	0.4	5.8	12.9	38.7	27.1	15.1		0.4	10.3	16.4	36.0	24.4	12.5		ns
DH-Snacking – fruit	2	18.7	81.3						13.3	86.7						ns
DH-Snacking – vegetables	2	51.6	48.4						41.3	58.7						ns
DH-Snacking – unsweetened dairy beverages and desserts	2	64.9	35.11						67.6	32.4						ns
DH-Snacking – sweetened dairy beverages and desserts	2	83.6	16.4						79.6	20.4						ns
DH-Snacking – sweet snacks	2	30.2	69.8						34.7	65.3						ns
DH-Snacking – savoury snacks	2	55.6	44.4						52.0	48.0						ns
DH-Snacking – nuts, almonds, seeds	2	46.7	53.3						49.8	50.2						ns
DH-Type of milk and dairy beverages (by fat content)	3	42.3	53.2	4.5					41.5	54.0	4.5					ns
DH-Type of heat treatment of meat – boiled	2	73.4	28.6						67.9	32.1						ns
DH-Type of heat treatment of meat – stewed	2	92.9	7.1						92.9	7.1						ns

DH-Type of heat treatment of meat – grilled	2	58.0	41.5						60.7	39.3						ns
DH-Type of heat treatment of meat – roasted	2	72.2	27.8						68.8	31.2						ns
DH-Type of heat treatment of meat – fried	2	20.1	79.9						23.8	76.2						ns
DH-Type of heat treatment of meat – I don't eat meat	2	84.4	15.6						84.4	15.6						ns
DH-Type of bread spread	7	0.5	21.6	11.9	0.9	12.4	45.4	7.3	19.1	18.1	0.9	10.7	42.8	7.9	0.5	ns
DH-Type of frying fat	6	2.3	21.1	65.9	5.1	5.6	-		4.7	20.9	62.6	3.8	7.6	0.4		ns
DH-Sweetening hot beverages	4	76.9	16.0	4.0	3.1				74.1	20.9	1.8	3.1				ns
DH-Adding salt to meals	3	71.11	20.9	8.0					69.2	23.2	7.6					ns
DH-Type of water consumed – I don't drink water	2	99.6	0.4						98.7	1.3						ns
DH-Type of water consumed – still water	2	20.4	79.6						21.3	78.7						ns
DH-Type of water consumed – sparkling water	2	61.8	38.2						64.9	35.1						ns
DH-Type of water consumed – flavoured water	2	88.0	12.0						87.1	12.9						ns
FF-Vegetable oils, margarines, mixes of butter and margarines	6	32.5	29.4	18.8	15.7	3.0	0.5		32.5	28.4	19.8	16.8	1.5	1.0		ns
FF-Eggs	6	5.6	27.9	31.5	31.0	3.6	5		7.1	27.9	27.9	33.5	3.0	0.5		ns
FF-Potatoes (excluding chips and crisps)	6	0.5	12.7	28.4	55.8	2.5	0.0		1.5	13.7	28.4	52.8	2.5	1.0		ns
FF-Instant soups, ready-made soups	6	35.0	37.1	12.2	11.7	3.6	0.5		33.5	37.6	12.2	12.2	4.6	0.0		ns
FF-Tinned vegetables	6	29.4	56.3	8.1	6.1	0.0	0.0		32.0	49.7	11.2	6.6	0.5	0.0		ns

FF-Fruit juices	6	12.2	40.6	21.3	20.3	3.0	2.5		9.6	43.7	21.3	19.8	4.1	1.5		ns
FF-Vegetable juices, fruit and vegetable juices	6	26.4	45.2	16.2	11.7	0.5	0.0		28.9	40.1	19.3	10.7	1.0	0.0		ns
FF-Sweetened hot beverages	6	6.6	11.7	11.2	25.9	24.9	19.8		5.6	6.6	10.7	30.5	21.3	25.4		0.05
FF-Water	6	0.5	4.6	8.6	16.2	9.1	60.9		0.5	5.1	5.6	14.7	7.6	66.5		ns
LPD-Eating out	5	3.2	50.2	22.6	19.0	5.0			3.7	53.4	20.2	18.3	4.6			ns
LPD-Type of alcohol usually consumed	4	39.3	28.0	23.1	9.7				36.0	31.7	22.0	10.3				ns
LPD-Smoking currently	2	79.6	20.4						80.7	19.3						ns
LPD-Smoked in the past	2	63.1	36.9						61.9	38.1						ns
LPD-Sleep time on weekdays	3	19.6	77.3	3.1					21.2	75.2	3.6					ns
LPD-Sleep time on weekends	3	4.4	57.4	38.2					5.4	56.0	38.6					ns
LPD-Time spend watching TV or using a computer	5	7.1	26.2	35.6	24.4	3.6	3.1		4.5	26.6	35.1	24.3	8.1	1.4		ns
LPD-Physical activity at work/school	4	53.8	42.2	3.6	0.4				50.0	48.2	1.8					ns
LPD-Physical activity during leisure time	3	7.1	47.1	45.8					9.9	52.5	37.7					0.01
LPD-Self-assessment of health status compared to other people of the same age	3	9.3	72.9	17.8					9.4	73.1	17.5					ns
LPD-Self-assessment of nutrition knowledge	4	24.4	44.0	26.7	4.9				20.6	51.1	25.1	3.2				ns
LPD-Self-assessment of diet	4	1.3	26.3	68.8	3.6				2.3	24.3	71.2	2.2				ns
LPD-Self-assessment of diet during weekdays compared to weekends	3	26.6	59.6	13.8					25.3	64.7	10.0					ns

DH – dietary habits; FF – food frequency; LPD – lifestyle and personal data; ns: not statistically significant; p: significance level: 0.05; 0.01; 0.001; <0.001;

Table S2. Nutrition questionnaire according to the number of categories of responses in students from Slovakia (%).

Questions	N°	Test							Retest							p
		1	2	3	4	5	6	7	1	2	3	4	5	6	7	
DH-Number of meals a day	5	2.5	6.6	28.4	43.2	19.3			1.0	5.1	37.1	38.1	18.2	0.5		ns
DH-Regularity of consuming meals	3	45.6	50.8	3.6					42.6	54.3	3.1					ns
DH-Frequency of snacking between meals	6	1.5	4.1	8.6	26.9	32.0	27.9		2.0	7.6	12.7	27.4	31.5	18.8		< 0.001
DH-Snacking – fruit	2	22.8	77.2						19.8	80.2						ns
DH-Snacking – vegetables	2	64.5	35.5						51.8	48.2						< 0.001
DH-Snacking – unsweetened dairy beverages and desserts	2	64.5	35.5						69.0	31.0						ns
DH-Snacking – sweetened dairy beverages and desserts	2	72.1	27.9						77.7	22.3						ns
DH-Snacking – sweet snacks	2	80.2	19.8						72.6	27.4						ns
DH-Snacking – savoury snacks	2	76.7	23.3						81.7	18.3						0.01
DH-Snacking – nuts, almonds, seeds	2	71.1	28.9						74.6	25.4						ns
DH-Type of milk and dairy beverages (by fat content)	3	62.9	35.1	2.0					64.0	33.5	2.5					ns
DH-Type of heat treatment of meat – boiled	2	23.4	76.6						19.8	80.2						ns

DH-Type of heat treatment of meat – stewed	2	39.6	60.4						35.5	64.5						ns
DH-Type of heat treatment of meat – grilled	2	60.9	39.1						53.3	48.7						0.01
DH-Type of heat treatment of meat – roasted	2	30.0	70.0						31.5	68.5						ns
DH-Type of heat treatment of meat – fried	2	59.9	40.1						59.9	40.1						ns
DH-Type of heat treatment of meat – I don't eat meat	2	98.0	2.0						96.4	3.6						ns
DH-Type of bread spread	7	13.2	6.6	0.5	1.1	75.1	2.5	1.0	14.7	6.6	2.0	2.0	69.5	3.1	2.1	ns
DH-Type of frying fat	6	2.5	6.1	76.7	0.5	6.1	8.1		1.0	9.6	76.1	7.1	6.2			ns
DH-Sweetening hot beverages	4	35.5	35.5	29.0					36.0	36.6	27.4					ns
DH-Adding salt to meals	3	46.2	45.7	8.1					47.2	45.7	7.1					ns
DH-Type of water consumed – I don't drink water	2	98.5	1.5						99.0	1.0						ns
DH-Type of water consumed – still water	2	35.0	65.0						34.0	66.0						ns
DH-Type of water consumed – sparkling water	2	68.0	33.0						69.5	30.5						ns
DH-Type of water consumed – flavoured water	2	70.6	29.4						73.6	26.4						ns
FF-Vegetable oils, margarines, mixes of butter and margarines	6	32.5	28.4	19.8	16.8	1.5	1.0		32.5	29.4	18.8	15.7	3.1	0.5		ns
FF-Eggs	6	7.1	27.9	27.9	33.5	3.1	0.5		5.6	27.9	31.5	31.0	3.5	0.5		ns
FF-Potatoes (excluding chips and crisps)	6	1.5	13.7	28.4	52.8	2.5	1.0		0.5	12.7	28.4	55.5	2.5			ns
FF-Instant soups, ready-made soups	6	33.5	37.6	12.2	12.2	4.6	-		35.0	37.1	12.2	11.7	3.6	0.5		ns

FF-Tinned vegetables	6	32.0	49.7	11.2	6.6	0.5	-		29.4	56.4	8.1	6.1				ns
FF-Fruit juices	6	9.6	43.7	21.3	19.8	4.1	1.5		12.2	40.6	21.3	20.3	3.1	2.5		ns
FF-Vegetable juices, fruit and vegetable juices	6	29.4	40.1	18.8	10.7	1.0	-		26.4	45.2	16.2	11.7	0.5			ns
FF-Sweetened hot beverages	6	5.6	6.6	10.7	30.5	21.3	25.4		6.6	11.7	11.2	25.9	24.9	19.8		0.05
FF-Water	6	0.5	5.1	5.6	14.7	7.6	66.5		0.5	4.6	8.6	16.2	9.1	60.9		ns
LPD-Eating out	5	3.6	45.2	29.4	19.3	2.0	0.5		5.58	46.70	25.38	19.80	2.54			ns
LPD-Type of alcohol usually consumed	5	23.4	37.1	10.2	7.0	22.3			22.8	39.6	9.6	6.6	21.4			ns
LPD-Smoking currently	2	77.7	22.3						77.2	22.8						ns
LPD-Smoked in the past	2	64.0	36.0						66.5	33.5						ns
LPD-Sleep time on weekdays	3	36.0	59.9	4.1					37.1	59.4	3.5					ns
LPD-Sleep time on weekends	3	12.18	53.30	34.52					13.2	52.3	34.5					ns
LPD-Time spend watching TV or using a computer	5	32.0	37.6	20.8	8.1	0.5	1.0		34.0	38.6	17.3	8.1	1.0	1.0		ns
LPD-Physical activity at work/school	4	25.9	65.0	9.14					23.9	69.0	7.1					ns
LPD-Physical activity during leisure time	3	21.32	65.48	13.20					18.3	72.6	9.1					ns
LPD-Self-assessment of health status compared to other people of the same age	3	13.7	71.1	15.2					12.2	74.1	13.7					ns
LPD-Self-assessment of nutrition knowledge	4	8.6	44.2	41.6	5.6				8.1	42.1	6.1					ns
LPD-Self-assessment of diet	4	3.1	26.4	2.5					1.5	26.0	1.0					ns

LPD-Self-assessment of diet during weekdays compared to weekends	3	34.5	50.8	14.7					31.5	52.8	15.7					ns

DH – dietary habits; FF – food frequency; LPD – lifestyle and personal data; ns: not statistically significant; p: significance level: 0.05; 0.01; 0.001; <0.001;
